# Genomic diversity of β-lactamase producing *Pseudomonas aeruginosa* in Iran; the impact of global high-risk clones

**DOI:** 10.1186/s12941-024-00668-5

**Published:** 2024-01-13

**Authors:** Nazila Ahmadi, Himen Salimizand, Abolfazl Rafati Zomorodi, Jalileh Ebn Abbas, Rashid Ramazanzadeh, Fakhri Haghi, Sepideh Hassanzadeh, Mojdeh Jahantigh, Mojtaba Shahin

**Affiliations:** 1 Student Research Committee, Kurdistan University of Medical Sciences, Sanandaj, Iran; 2 Cellular and Molecular Research Center, Research Institute for Health Development, Kurdistan University of Medical Sciences, Sanandaj, Iran; 3 Department of Bacteriology and Virology, School of Medicine, Shiraz University of Medical Sciences, Shiraz, Iran; 4 Department of Microbiology, Faculty of Medicine, Kurdistan University of Medical Sciences, Sanandaj, Iran; 5 Department of Microbiology, Faculty of Medicine, Ardabil University of Medical Sciences, Ardabil, Iran; 6 Department of Microbiology, Zanjan University of Medical Sciences, Zanjan, Iran; 7 Department of Microbiology and Virology, Faculty of Medicine, Mashhad University of Medical Sciences, Mashhad, Iran; 8 Department of Microbiology, School of Medicine, Zahedan University of Medical Sciences, Zahedan, Iran; 9 Department of Medical Laboratory Sciences, Faculty of Medical Sciences, Arak branch, Islamic Azad University, Arak, Iran

**Keywords:** *Pseudomonas aeruginosa*, MLST, High-risk clones, Plasmid-borne *ampC*, ESBLs, Carbapenemase

## Abstract

**Background:**

Hospital-acquired infections caused by multidrug-resistant *Pseudomonas aeruginosa* incline hospital stay and costs of treatment that resulted in an increased mortality rate. The frequency of *P. aeruginosa* high-risk clones producing carbapenemases was investigated in our clinical samples.

**Methods:**

In this cross-sectional study, 155 non-repetitive *P. aeruginosa* isolates were included from different medical centers of Iran. Antibiotic susceptibility testing was determined, and the presence of β-lactamases were sought by phenotypic and genotypic methods. The clonal relationship of all isolates was investigated, and multi-locus sequence typing (MLST) was used for finding the sequence types of carbapenemase-producers.

**Results:**

The agent with highest percent susceptibility rate was recorded for colistin (94.9%). MOX and FOX were found both as low as 1.95% (3/155). The most frequent narrow spectrum β-lactamase was SHV with 7.7% (12/155) followed by PER, OXA-1, and TEM with the frequency of 7.1% (11/155), 3.2% (5/155), and 1.3% (2/155), respectively. Carbapenemases were detected in 28 isolates (18%). The most frequent carbapenemase was IMP with 9% (14/155) followed by NDM, 8.4% (13/155). OXA-48 and VIM were also detected both per one isolate (0.65%). MLST of carbapenem resistant *P. aeruginosa* isolates revealed that ST244, ST664, ST235, and ST357 were spread in subjected clinical settings. REP-PCR uncovered high genomic diversity in our clinical setting.

**Conclusion:**

Clonal proliferation of ST235 strain plays a key role in the propagation of MDR pattern in *P. aeruginosa*. Our data showed that high-risk clones has distributed in Iran, and programs are required to limit spreading of these clones.

## Introduction

*Pseudomonas aeruginosa* is an important cause of hospital-acquired infections, and immune-deficient patients such as neutropenia, burnt patients, and cystic fibrosis [1]. *P. aeruginosa* is intrinsically resistant to various antibiotics due to low permeability of the outer membrane, production of chromosomal β-lactamases, and the efflux pumps [2, 3]. Therefore, appropriate drug choice for *P. aeruginosa* infections are limited to the combination of aminoglycosides, fluoroquinolones, carbapenems, β-lactam/inhibitors, and colistin [1].

Studies have shown that mortality due to infection with metallo-β-lactamase (MBL) producing *P. aeruginosa* is much higher than MBL-negative strains [4]. Frequently reported MBLs included VIM and IMP, and recently NDM-1 variants [5]. The integron-mediated IMP and VIM β-lactamases are currently the most widespread genes reported from several continents with diverse genotypes [6].

β-lactamases are transmitted in various ways from one bacterium to another that plays a key role in the epidemiology of antimicrobial resistance. Molecular typing methods such as Repetitive-Extragenic Palindromic PCR (REP-PCR) and Multi-Locus Sequence Typing (MLST) are used to study the relationship of isolates in health-care centers [7].

Tracing multidrug resistant clones are crucial for infection control to avoid detrimental consequences of antimicrobial resistance. Several *P. aeruginosa* high-risk clones (PAHRC) are currently circulating globally in the healthcare centers including ST175, ST235, and ST111 [8]. PAHRC refers to clones of *P. aeruginosa* that caused multiple epidemic outbreaks in different hospital centers worldwide. They are mostly MDR or XDR and highly virulent but with low genomic diversity. There are several reports of PAHRC outbreaks in various countries, however, information of epidemiology of these clones are very rare in the eastern countries of Asia. Furthermore, an update about recent in-circulate clones is of interest. In this study, the phylogenetic relationship, antimicrobial susceptibility profile, and the epidemiology of PAHRCs in β-lactamases producing *P. aeruginosa* isolates from different medical centers in Iran was studied.

## Materials and methods

### Sample collection

This prospective cross-sectional multicenter study was started on July 2018 to the end of June 2019 at six hospitals centers across the country, Iran, including Sanandaj, Mashhad, Zanjan, Zahedan, Ahvaz, and Hamedan. Hospitalized and outpatients as well as healthcare-associated equipment (HAE) samples were considered for this study. One isolate per patient was included. Biochemical reactions including growth on MacConkey agar, oxidase reaction, sugar consumption in TSI medium, Oxidation-Fermentation (OF) activity, mobility, and growth at 42 °C were assessed to isolate *Pseudomonas* species.

PCR was used to species detection. DNA was extracted by boiling and 5΄-CCTGACCATCCGTCGCCACAAC-3΄ and 5΄-CGCAGCAGGATGCCGACGCC-3΄ primers were used [9]. The PCR process was performed, and PCR products were run in 1.5% agarose gel. The 222-bp band represented the target *P. aeruginosa*. *P. aeruginosa* ATCC 27,853 was used as a positive control in all experiments.

### Antimicrobial susceptibility testing

The antibiotic resistance profile of *P. aeruginosa* isolates was determined by Kirby-Bauer disk diffusion method on Müller-Hinton agar (Himedia, India) and the results were interpreted according to the Clinical Laboratory Standards Institute (CLSI) criteria [10]. Colistin (10µgr), meropenem (10µgr), ceftazidime (30µgr), ciprofloxacin (5µgr), levofloxacin (5µgr), tobramycin (10µgr), amikacin (30µgr), gentamicin (10µgr), and piperacillin (100µgr) (BD, Sparks, MD, USA) were considered for this study. Colistin broth disk elution (CBDE) method was performed for colistin susceptibility [11]. *P. aeruginosa* ATCC 27,853 standard was used as quality control. Multidrug resistance (MDR), extensively drug resistance (XDR), and pan drug resistance (PDR) patterns were defined based on Magiorakos criteria [12]. According to the criteria, it is defined that MDR is acquired non-susceptibility to at least one agent belonging to three or more antimicrobial categories. As defined by XDR, it is non-susceptibility to all antimicrobial categories except two or fewer (i.e., bacterial isolates remain susceptible to only one or two antimicrobial categories), and as defined by PDR, it is non-susceptibility to all antimicrobial agents.

### Phenotypic detection of β-lactamases

β-lactam/inhibitor (BLI) combined disks including ceftazidime/clavulanic acid and cefepime/clavulanic acid were considered for isolates that showed resistance phenotype to cefepime and ceftazidime. On the other hand, isolates that demonstrated carbapenem resistant or intermediate phenotype in susceptibility testing were subjected for modified carbapenem inactivation method (mCIM) [10, 13]. mCIM is only valid in Enterobacterials and *P. aeruginosa*. All the cephalosporin resistant isolates, regardless of positive combined disk result, as well as mCIM positive isolates, were further investigated to find β-lactamase genes by molecular PCR assay.

### Molecular detection of β-lactamases

To detect β-lactamase genes, multiplex PCR was done, as previously described [14–17]. The list of primers including narrow-spectrum β-lactamases (NSBL), ESBLs, plasmid-borne *ampC* (PB*ampC*), and carbapenemases are presented in Table 1. Ready-to-use MasterMix (Parstous, Iran) was used in 25 µl reaction. Strains with favorable genes in our repertoire that were collected from previous studies considered as positive control [18, 19]. Those without positive control in our collection, were sequenced through Sanger sequencing by ABI 3730XL sequencer. PCR products were run in 1.5% agarose and the gels were examined using ethidium bromide and visualized by UV.

**Table 1:** Primers used in this study

Genes	Primer sequence	Cycling Conditions	Product length (bp)	Reference
*blaOXA-48*	GCGTGGTTAAGGATGAACAC CATCAAGTTCAACCCAACCG	94 °C-10΄, 36 cycles of 94 °C-30˝, 52 °C-40˝, 72 °C-50˝, with 72 °C-5΄	438	[17]
*blaIMP*	GAAGGYGTTTATGTTCATAC GTAMGTTTCAAGAGTGATGC		232	[17]
*blaVIM*	GTTTGGTCGCATATCGCAAC AATGCGCAGCACCAGGATAG		390	[17]
*blaNDM*	GGTTTGGCGATCTGGTTTTC CGGAATGGCTCATCACGATC		621	[17]
*blaGES*	AGTCGGCTAGACCGGAAA GTTTGTCCGTGCTCAGGAT		399	[17]
*blaSPM*	AAAATCTGGGTACGCAAACG ACATTATCCGCTGGAACAGG		271	[17]
*blaDIM*	GCTTGTCTTCGCTTGCTAACG CGTTCGGCTGGATTGATTTG		699	[17]
*blaBIC*	TATGCAGCTCCTTTAAGGGC TCATTGGCGGTGCCGTACAC		537	[17]
*blaSIM*	TACAAGGGATTCGGCATCG TAATGGCCTGTTCCCATGTG		570	[17]
*blaGIM*	TCGACACACCTTGGTCTGAA AACTTCCAACTTTGCCATGC		477	[17]
*blaAIM*	CTGAAGGTGTACGGAAACAC GTTCGGCCACCTCGAATTG		322	[17]
TEM	CATTTCCGTGTCGCCCTTATTC CGTTCATCCATAGTTGCCTGAC	94 °C-10΄, 30 cycles of 94 °C-40˝, 60 °C-40˝, 72 °C-60˝, with 72 °C-7΄	800	[14]
SHV	AGCCGCTTGAGCAAATTAAAC ATCCCGCAGATAAATCACCAC		713	[14]
OXA-1	GGCACCAGATTCAACTTTCAAG GACCCCAAGTTTCCTGTAAGTG		564	[14]
CTX-M group 1	TTAGGAARTGTGCCGCTGYA CGATATCGTTGGTGGTRCCAT		688	[14]
CTX-M group 2	CGTTAACGGCACGATGAC CGATATCGTTGGTGGTRCCAT		404	[14]
CTX-M group 8	AACRCRCAGACGCTCTAC TCGAGCCGGAASGTGTYAT		326	[14]
CTX-M group 9	TCAAGCCTGCCGATCTGGT TGATTCTCGCCGCTGAAG		561	[14]
ACC	AACAGCCTCAGCAGCCGGTTA TTCGCCGCAATCATCCCTAGC	94 °C-3΄, 25 cycles of 94 °C-30˝, 64 °C-30˝, 72 °C-60˝, with 72 °C-7΄	346	[16]
ACT1-MIR1	TCGGTAAAGCCGATGTTG CGG CTTCCACTGCGGCTGCCAGTT		302	[16]
FOX	AACATGGGGTATCAGGGAGATG CAAAGCGCGTAACCGGATTGG		190	[16]
MOX	GCTGCTCAAGGAGCACAGGAT CACATTGACATAGGT GTGGTGC		520	[16]
LAT	TGGCCAGAACTGACAGGCAAA TTTCTCCTGAACGTGGCTGGC		462	[16]
DHA	AACTTTCACAGGTGTGCTGGGT CCGTACGCATACTGGCTTTGC		405	[16]
acsA*	GCCACACCTACATCGTCTAT GTGGACAACCTCGGCAACCT	94 °C-3΄, 35 cycles of 96 °C-60˝, 55 °C-60˝, 72 °C-60˝, with 72 °C-7΄	390	[20]
aroE*	ATGTCACCGTGCCGTTCAAG TGAAGGCAGTCGGTTCCTTG		495	[20]
guaA*	AGGTCGGTTCCTCCAAGGTC TCAAGTCGCACCACAACGTC		372	[20]
mutL*	AGAAGACCGAGTTCGACCAT ATGACTTCCTCTATGGCACC		441	[20]
nuoD*	ACGGCGAGAACGAGGACTAC TTCACCTTCACCGACCGCCA		366	[20]
ppsA*	GGTGACGACGGCAAGCTGTA TCCTGTGCCGAAGGCGATAC		369	[20]
trpE*	TTCAACTTCGGCGACTTCCA GGTGTCCATGTTGCCGTTCC		441	[20]

^*Primers used for multi−locus sequence typing (MLST)^

### Genetic relationship of isolates

To find the genomic relatedness between and amongst the isolates, REP-PCR was performed, as previously described [21]. Captured pictures were analyzed by GelJ (v. 1.3) software with dice tolerance 2.0 and UPGMA method to draw the dendrogram. To find clusters, 80% of similarity was considered [21].

### Multi-locus sequence typing

MLST by seven housekeeping genes was performed for those isolates that were carbapenem-resistant [20]. PCR was performed on the extracted DNA of the isolates and PCR products were sequenced. The obtained sequences were embedded at www.pubmlst.org to identify the desired alleles and the sequence type of the strains was finally determined.

## Results

### Bacterial isolates

From five medical centers of Iran 155 samples were collected. Hospitals were in different districts. The highest frequency of *P. aeruginosa* was collected from burn ward (40.4%) followed by ICU (25%), Infection (15.6%), outpatients (16.7%), and healthcare associated instruments (2.3%). Isolates were mainly from wound samples with a frequency of 57.3% followed by tracheal aspirates (20.1%), blood (11%), urine (9.7%), and HAE (1.9%).

### Antimicrobial susceptibility profile

According to the results of antimicrobial susceptibility testing, the most effective chemical was colistin, to which 94.9% (147/155) of the isolates were susceptible. Subsequently, meropenem with 70.9% (110/155) and piperacillin with 60.6% (94/155) were effective on isolates. Furthermore, the most resistance rate was related to ciprofloxacin (34.8%) followed by cefepime (50.3%) (Table 2). Regarding Magiorakos et al. criteria, 2.6% of isolates were PDR (4/155), 25.1% were XDR (139/55), and 16.8% were MDR (26/155).

**Table 2:** Antimicrobial susceptibility testing of 155 *P. aeruginosa* isolates (%)

	Susceptibility testing		
Antibacterial	Susceptible (%)	Intermediate (%)	Resistant (%)
Colistin §	147 (94.9)	0	8 (5.1)
Meropenem	110 (70.9)	9 (5.8)	39 (25.1)
Piperacillin	94 (60.6)	20 (12.9)	41 (26.5)
Amikacin	93 (60)	4 (2.6)	58 (37.4)
Levofloxacin	93 (60)	17 (10.9)	76 (49.1)
Tobramycin	86 (55.5)	5 (3.2)	64 (41.3)
Gentamicin	84 (54.2)	6 (3.8)	65 (41.9)
Ceftazidime	81 (52.2)	0 (0)	74 (47.7)
Cefepime	78 (50.3)	3 (1.9)	74 (47.7)
Ciprofloxacin †	54 (34.8)	25 (16.1)	76 (49.1)

^§ The highest susceptible; Susceptibility for colistin was checked by Colistin broth disk elution (CBDE) method^

^† The least effective antibiotic^

### Phenotypic detection of carbapenemase and ESBL-producing isolates

In the screening test for ESBLs performed on ceftazidime and/or cefepime resistant isolates, 11% (17/155) were indicated as ESBL-producer. Regarding phenotypic MBL test performed on carbapenem-resistant isolates, 28 out of 39 meropenem-resistant isolates were identified in mCIM while 11 isolates did not react in this test. It means that mCIM positive isolates had 6–15 mm zone diameter around the meropenem disk. Carbapenemase producing isolates hydrolyze the meropenem content on disk while suspending it in MHB media, and subsequent transfer of this disk on a plate inoculated with meropenem-susceptible *E. coli* ATCC 25,922 resulted in no inhibition growth.

### Molecular identification β-lactamase genes

Β-lactamases, rather than carbapenemase genes, were amplified in 19.3% (30/155) cephalosporin resistant isolates. The most frequent was SHV with 7.7% (12/155) followed by PER, OXA-1, and TEM with the frequency of 7.1% (11/155), 3.2% (5/155), and 1.3% (2/155), respectively. Regarding PB*ampC*, MOX and FOX were found both as low as 1.95% (3/155).

Carbapenemase multiplex-PCR on CRPA isolates revealed that 28 isolates (18%) harbored carbapenemase gene. The highest frequency of these genes was related to IMP with 9% (14/155) followed by NDM, 8.4% (13/155). OXA-48 and VIM were also detected per one isolate (0.65%). Other β-lactamases including SPM, SIM, GIM, BIC, DIM, AIM, GES, CTX-M-1 to CTX-M-9, DHA, ACC, MOX, and MIR1 genes were not found. In one CRPA isolate, NDM and OXA-48 genes were found simultaneously (Table 3).

**Table 3:** Frequency of *P. aeruginosa* producing β-lactamases (*N* = 155)

β-lactamases	Number of isolates	Frequency
IMP	14	9%
NDM	13	8.4%
OXA-48	1	0.65%
VIM	1	0.65%
SHV	12	7.7%
PER	11	7.1%
OXA-1	5	3.2%
TEM	2	1.3%
LAT	3	1.95%
FOX	3	1.95%

### Sequence type distribution of carbapenem resistant P. aeruginosa isolates

MLST for 39 CRPA isolates was performed, and it was revealed that the most prevalent was ST664 with the frequency of 12 isolates. ST235, ST244, and ST357, of each seven strains, were also distributed in subjected centers. ST150 and ST57 were observed as low frequent as two cases each, in a center.

### Clonality and genomic relationship

**Fig. 1:**
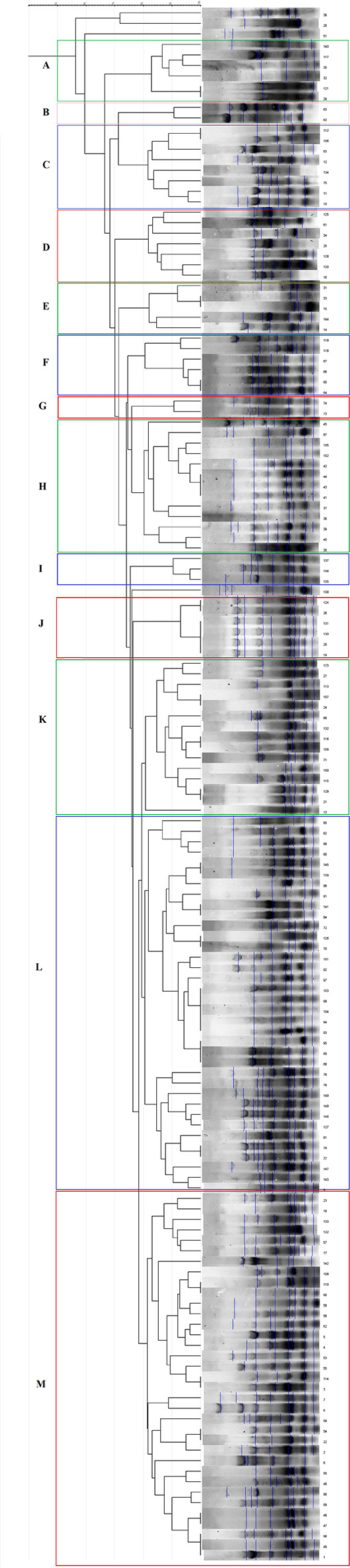
**1:** Dendrogram of REP-PCR analysis of 148 *P. aeruginosa* isolates drew with tolerance **2:** Seven isolates did not present any band in REP-PCR experiment. Clusters are shown in color boxes

Generated dendrogram showed that most of isolates categorized in 13 clusters (Fig. 1). However, four isolates were singleton, and seven isolates did not produce any bands. REP-PCR could not differentiate CRPA strains from non-MBL producers. The most populated cluster was L with 36 members followed by M cluster with 35. The most homogenous cluster was J-cluster that all strains were closely related and belonged to a burn medical center. Excluding J-cluster, other clusters showed high diversity even for strains isolated from the same wards of a center, at the close timeline of collecting. Low rate of clonality and wide genomic diversity was observed in all centers. CRPA as well as β-lactamase producing strains were distributed in different clusters.

## Discussion

The present study was performed to determine the genetic diversity of high-risk *P. aeruginosa* clones in Iran. *P. aeruginosa* infections have been reported with a mortality rate of 23% in different parts of the world [22]. A meta-analysis study by Mohammadpour et al. in Iran showed that out of 7548 isolates of *P. aeruginosa*, the overall prevalence of MBL genes was 16% that the most prevalent was VIM and with 6% of mortality [4]. Although VIM was detected in as low as 0.65% of our studied isolates, the main and important difference of our study was the increment prevalence of NDM with 8.4% of frequency. On the other hand, previous reports on CRPA from different countries indicated that carbapenem resistance was often associated with VIM and IMP rather than NDM [23, 24]. It should be mentioned that the emergence of NDM producing *P. aeruginosa* was also reported in other countries that isolates belonged to different sequence types [25, 26].

ST235 (belong to CC235) as the most distributed HRC has been associated with PER, OXA, and VIM. ST235 has also been identified in Spain as the GES carrier [27]. In the present study, carbapenem-resistant ST235 strains was also detected in most centers of Iran. Furthermore, the XDR pattern and the OXA-48 gene were detected exclusively in ST235, indicating clonal expansion of this strain in the studied centers. This clone had been previously highlighted in this region [28]. CRPA isolates from South America (Colombia) were associated with ST111 and ST235 that carried *bla*_VIM_ and *bla*_KPC_, respectively [29]. *P. aeruginosa* belong to these STs has also been reported in the UK that had been MBL producer [30]. ST235 is associated with serotype O11 which is the most prevalent serotype globally. Moreover, the presence of specific variant of DprA enzyme enhances the durability of antimicrobial resistance elements in the bacterium that helps colonization and distribution of this clone [31]. This enzyme is involved in homologous recombination.

Genotypic detection of NSBL and ESBLs revealed 19.7% (30/155) of *P. aeruginosa* isolates carried these variants while phenotypic ESBL was positive in 11% (17/155) of isolates. Discrepancy between these two methods can be explained by the co-presence of MBLs with ESBLs such that these enzymes had been overlooked by MBLs [23].

The function of intrinsic inducible AmpC has been perfectly studied in *P. aeruginosa* and it has been revealed how this bacterium react when dealt with β-lactamases [32]. However, information regarding PB*ampC* distribution in *P. aeruginosa* is limited. The current study showed that narrow range of *P. aeruginosa* harbored PB*ampC*. A study by Zhu et al. showed higher rate of these type of β-lactamases in their clinical setting [33]. It has been also investigated in non-clinical isolates of *P. aeruginosa* [34]. Low prevalence of this type of β-lactamases in *P. aeruginosa* compared to Enterobacteriaceae shows the lower potency of *P. aeruginosa* plasmids to harbor PB*ampC* β-lactamases.

Managing emergence of PAHRC crisis requires a greater commitment to basic and clinical research on infection control and antimicrobial stewardship. Without these implications, controlling high-risk clones will be impossible and it results in higher morbidity and mortality. A limitation to this project that may enrich our knowledge regarding distribution of high-risk clones is the plasmids that carry the carbapenemase genes. Future studies will focus on this issue.

## Conclusion

We found that different HRC of CRPA are in circulate in different hospitals of Iran. Colistin remained as a good drug choice with low resistance rate. There was a vast genetic diversity with a low ESBLs and PB*ampC* carriage in our centers. Hospital centers must pursue basic measures to control the infection, and further antibiotic monitoring is required.

## Data Availability

The datasets used and/or analysed during the current study are available from the corresponding author on reasonable request.
